# Structural basis of the oligomerization of anti-silencer Ler from enterohemorrhagic *E. coli* (EHEC)

**DOI:** 10.1063/4.0001203

**Published:** 2026-04-14

**Authors:** Guanghao Wang, Bo Duan, Bin Xia

**Affiliations:** 1 Beijing Nuclear Magnetic Resonance Center, Peking University, Beijing 100871, China; 2 School of Life Sciences, Peking University, Beijing 100871, China; 3 Suzhou Key Laboratory of Pathogen Bioscience and Anti-infective Medicine, MOE Key Laboratory of Geriatric Diseases and Immunology, Institute of Molecular Enzymology & School of Biology and Basic Medical Sciences, Soochow University, Suzhou 215123, China; 4 College of Chemistry and Molecular Engineering, Peking University, Beijing 100871, China

## Abstract

The locus of enterocyte effacement-encoded regulator (Ler) is a master transcriptional activator essential for the virulence of enterohemorrhagic and enteropathogenic *Escherichia coli*. Although Ler shares homology with the global silencer H-NS, it functions uniquely as an anti-silencer, a role strictly dependent on its oligomerization state. However, the structural mechanism governing Ler assembly remains poorly understood. In this study, we have characterized the N-terminal oligomerization domain (Ler^1–74^) of Ler using solution NMR spectroscopy and biophysical assays, and found that Ler^1–74^ shows concentration-dependent oligomerization. We demonstrate that Ler oligomerization is driven by two distinct interfaces with contrasting dynamic properties. We determined the solution structure of the Ler^18–74^ dimer, revealing a stable, anti-parallel “tail-to-tail” interface (dimer Site-2, residues 35–66) stabilized by a hydrophobic core. In contrast, the N-terminal interface (dimer Site-1, residues 12–33) forms a highly dynamic “head-to-head” dimer, which undergoes significant conformational exchange and exhibits concentration- and temperature-dependent dimerization. Based on these findings, we propose a structural model wherein Ler forms supramolecular assemblies through the propagation of alternating stable (Site-2) and dynamic (Site-1) interactions. This architecture, while reminiscent of H-NS, displays distinct stability features that may underlie Ler's specific anti-silencing function in bacterial pathogenesis.

## I. INTRODUCTION

The locus of enterocyte effacement-encoded regulator (Ler) is a critical transcriptional regulator in pathogenic strains of *Escherichia coli*, specifically enteropathogenic *E. coli* (EPEC) and enterohemorrhagic *E. coli* (EHEC), the pathogens responsible for severe gastrointestinal infections, including watery diarrhea, hemorrhagic colitis, and complications such as hemolytic uremic syndrome.^1,2^ Ler is encoded by the first gene within the LEE1 operon of the LEE pathogenicity island which encodes components of the type III secretion system (T3SS), translocators, effectors, and adhesins.^3^ There are multiple LEE operons (LEE1–LEE5), all of which are repressed by the histone-like nucleoid-structuring protein (H-NS), a global xenogeneic silencer of horizontally acquired AT-rich DNA sequences.^3^ Ler acts as a master activator of virulence genes by counteracting the repressive effects of H-NS on LEE2–LEE5 operons, thereby stimulating their expression and enabling bacterial colonization and host cell manipulation under virulence-inducing conditions.^4,5^ However, Ler can also auto-represses its own expression by downregulating LEE1 operon.^6,7^

Ler is a small protein of 123 amino acid residues and shares homology with H-NS particularly in the C-terminal DNA-binding domain (Fig. S1), but functions oppositely as an anti-silencing protein.^2,4^ This functional divergence is largely attributed to differences in oligomerization, a process where Ler forms multimers that influence DNA binding, complex formation, and gene activation.^8,9^ Previous studies suggested that Ler exhibits robust oligomerization in solution, forming dimers, higher-order multimers, and large soluble aggregates.^8^ Size-exclusion chromatography with multi-angle laser light scattering (SEC-MALS) analysis showed that Ler primarily exists as aggregates equivalent to 100–250 monomers at concentrations of 0.4–1.0 mg/mL, with less than 10% as low-order (2–10) multimers.^8^ However, atomic force microscopy (AFM) analysis of Ler revealed compact globular particles with multimodal volume distributions, corresponding mainly to oligomers of 4–5 or 15–20 monomers.^9^ The oligomerization of Ler is reported to be concentration-independent,^8^ unlike H-NS, which forms smaller oligomers in a concentration-dependent manner and binds DNA cooperatively depending on oligomerization.^8,10–12^

Predictions using the COILS program suggest that Ler possesses a N-terminal coiled-coil motif spans residues 10–40,^8^ and a central domain (residues 50–65) was found to be critical for its oligomerization.^13^ The C-terminal DNA binding domain covers residues 75–123, as revealed by its solution structure.^14^ Random mutagenesis of *ler* identified 18 different point mutations that derepress P_LEE1_ by 8- to 12-fold, all reducing DNA-binding affinity to <50% of wild-type levels. While most of these mutation sites are within the DNA binding domain, only L56P mutant failed in co-immunoprecipitation with His-tagged Ler, suggesting this mutation completely abolishes oligomerization of Ler.^13^ Truncation mutants further validated that residues 1–64 can form multimers but exerted dominant-negative effects, while residues 1–49 does not, pinpointing residues 50–64 as essential for oligomerization.^13^ Insertion of extra residues between residues 60 and 61 of Ler was found to impair higher-order oligomerization, increasing low-order multimers (30–70 kDa) and reducing DNA-binding efficiency.^8,15^

Despite the efforts in studying Ler through biochemical, biophysical, and genetic approaches, the molecular mechanism of the oligomerization of Ler is still unclear. Here, we report the structural characterization of the oligomerization domain of Ler. We demonstrate that Ler has two distinct dimerization interfaces and self-associates to form multimers through propagating the two “head-to-head” and “tail-to-tail” dimers, similar to that of the oligomerization domain of H-NS.^16^ We have determined the solution structure of Ler^18–74^, which contains the second and show a model structure demonstrating the propagating dimerization of the oligomerization domain of Ler.

## II. MATERIALS AND METHODS

### A. Protein production

The coding sequences of full-length Ler from enteropathogenic *E. coli* and its N-terminal truncation variant Ler^1–74^ were cloned into the pET-21a vector (Novagen) between *Nde*I and *Xho*I restriction sites, and thus, a C-terminal 6×His-tag was introduced. Coding sequences of other truncation variants (Ler^18–74^, Ler^1–36^, Ler^1–39^, and Ler^1–42^) were ligated into the pET-28a vector between the *Nde*I and *Xho*I sites, with an N-terminal 6×His-tag introduced, followed by a thrombin cleavage site.

The recombinant plasmids were transformed into *E. coli* BL21 (DE3) competent cells. For unlabeled protein expression, bacteria were initially cultured in 50 mL of Luria–Bertani (LB) medium at 37 °C until the optical density at 600 nm (*OD*_600_) reached 0.8. This starter culture was then transferred into 1 L of LB medium and grown until the *OD*_600_ reached 0.8. Expression of Ler and Ler^1–74^ was induced with 0.1 g/L IPTG at 37 °C for 6 h, whereas the expression of other constructs was induced at 25 °C for 24 h.

For isotope-labeled samples, bacteria were inoculated into 50 mL of ^15^N-labeled or ^15^N, ^13^C-double-labeled M9 minimal medium and then scaled up to a final volume of 500 ml. Upon reaching an *OD*_600_ of 0.8, protein expression was induced with 0.1 g/L IPTG using the same conditions as for the unlabeled proteins.

Bacteria were harvested by centrifugation and resuspended in 30 mL of lysis buffer (50 mM sodium phosphate, 1 M NaCl, 20 mM imidazole, pH 8.0). After sonication and clarification by centrifugation, the supernatant was loaded onto a Ni-NTA affinity column (Qiagen). The column was washed with lysis buffer, and the 6×His tagged proteins were eluted with elution buffer (50 mM sodium phosphate, 1 M NaCl, 250 mM imidazole, pH 8.0). For proteins expressed from the pET-28a vector, the N-terminal 6×His-tag was removed by digestion with two units of thrombin (Sigma) per mg protein at 25 °C for 5 h. The reaction mixture was then passed through a Ni-NTA column to remove the cleaved tag and uncleaved protein. Finally, all proteins were purified by size exclusion chromatography using HiLoad 16/600 Superdex 75 pg or HiLoad 16/600 Superdex 200 pg columns (GE Healthcare) in FPLC buffer (50 mM sodium phosphate, 50 mM NaCl, pH 7.0).

To prepare hybrid-labeled Ler^18–74^ dimer (comprising one unlabeled monomer and one uniformly ^15^N, ^13^C-labeled monomer), equimolar amounts of unlabeled and ^15^N, ^13^C-labeled Ler^18–74^ were mixed, and urea was added to a final concentration of 8 M. The mixture was gently rocked overnight at 4 °C. Refolding was initiated by slowly diluting the protein solution into a 100-fold volume of urea-free buffer. The sample was concentrated to 500 *μ*L, followed by buffer exchange to remove urea. Proper refolding was confirmed by 2D ^1^H-^15^N HSQC spectra.

### B. NMR experiments

All NMR samples were prepared in 50 mM sodium phosphate, 50 mM NaCl (pH 7.0), containing 95% H_2_O/5% D_2_O, supplemented with 0.01% sodium azide (NaN3), 0.01% DSS, and 0.02% protease inhibitor cocktail.

For the solution structure determination of Ler^18–74^, NMR data were acquired at 298 K. Resonance assignments and NOE restraints were derived from a 1.0 mM uniformly ^15^N, ^13^C-labeled sample. The following experiments were used for resonance assignment: 2D ^1^H-^15^N HSQC, 2D ^1^H-^13^C HSQC, 3D HNCA, 3D HN(CO)CA, 3D HNCACB, 3D CBCA(CO)NH, 3D HNCO, 3D HBHA(CO)NH, 3D (H)CCH-COSY, 3D (H)CCH-TOCSY, 3D H(C)CH-COSY. For structure determination, 3D ^1^H-^15^N NOESY-HSQC, 3D ^1^H-^13^C NOESY-HSQC (aliphatic), and 3D^1^H-^13^C aromatic NOESY-HSQC were collected with a mixing time of 120 ms. To detect intermolecular NOEs, 3D F1-^15^N/^13^C-filtered, F2-^13^C-edited NOESY-HSQC and 3D F1^−15^N/^13^C-filtered, F2-^15^N-edited NOESY-HSQC spectra were collected on the hybrid-labeled sample with a mixing time of 150 ms. Heteronuclear {^1^H}-^15^N NOE experiments were performed on a 0.5 mM ^15^N-labeled sample.

For other truncation variants, 2D ^1^H-^15^N HSQC spectra for Ler^1–36^ and Ler^1–39^ were acquired at 298 K using 0.2 mM samples. In the case of Ler^1–42^, 2D ^1^H-^15^N HSQC spectra were collected at temperatures ranging from 278 to 308 K with protein concentrations of 0.2–1.0 mM. Backbone assignments for Ler^1–42^ were performed at 278 K using a 1.0 mM uniformly ^15^N, ^13^C-labeled sample, and CEST experiments were conducted at 278 K using a 1.0 mM ^15^N-labeled sample with a saturation field strength *B*_1_ of 20 Hz.

NMR data were collected on Bruker AVANCE 500, 600, 700, or 950 MHz spectrometers equipped with cryogenic triple-resonance probes using standard Bruker pulse sequences. Proton chemical shifts were referenced directly to internal DSS, while ^15^N and ^13^C chemical shifts were referenced indirectly. The acquired NMR spectra were all processed using the NMRPipe^17^ and analyzed using NMRView.^18^

### C. Solution structure calculation

Structure determination of Ler^18–74^ utilized distance restraints derived from the NOESY datasets described above, combined with dihedral angle restraints and hydrogen bond restraints. Dihedral angle restraints included high-confidence predictions (88 PHI and 88 PSI angle restraints) generated by the TALOS-N program^19^ based on chemical shift assignments, along with an additional 28 empirical PHI angle restraints (0° to −180°). Hydrogen bond restraints (totaling 82 restraints; 41 per monomer) were added mainly based on secondary structures.

Intermolecular distance restraints were specifically obtained from the spectra of the hybrid-labeled sample. NOE assignments were assisted using SANE (structure-assisted NOE evaluation).^20^ An iterative SANE–CYANA cycle was employed for calculation: refined restraints from SANE were used to calculate structures using the torsion angle dynamics module of CYANA,^21,22^ and the resulting models were fed back into SANE to guide the next round of assignment. This cycle was repeated until no distance violations exceeded 0.2 Å. Subsequently, 1000 structures were calculated using CYANA. The 100 conformers with the lowest target function values were selected for further iterative refinement using AMBER 18^23^ and SANE.

The calculation was considered complete when over 95% of NOE restraints were utilized with no angular violations >5° and no distance violations >0.2 Å. The final ensemble consisted of the 20 structures with the lowest AMBER energy. Structure quality was validated using PROCHECK-NMR,^24^ and convergence was assessed using Suppose (part of the AMBER Tools) based on the method of Shapiro *et al.*^25^

### D. Analytical SEC

Analytical size-exclusion chromatography (SEC) was performed using a Superdex 200 Increase 10/300 GL column (GE Healthcare) connected to an AKTA FPLC system at room temperature. The column was equilibrated with a buffer containing 50 mM sodium phosphate and 50 mM NaCl (pH 7.0). Ler^1–74^ samples (100 *μ*L) at various concentrations were prepared in the same buffer and loaded onto the column at a flow rate of 0.5 mL/min. The elution profiles were monitored by absorbance at 280 nm. To calibrate the column, 100 *μ*L of bovine serum albumin (BSA) was analyzed under the same condition, which eluted as a mixture of monomer (66.4 kDa), dimer (132.8 kDa), trimer (199.2 kDa), and tetramer (265.6 kDa).

### E. Circular dichroism (CD)

The Ler^1–74^ sample was prepared at a concentration of 1.0 mg/mL in a buffer containing 50 mM sodium phosphate, 350 mM NaCl, and 0.2 mM EDTA (pH 7.0). For concentration-dependent analysis, Ler^1–42^ samples were prepared at 0.1, 0.2, 0.5, and 1.0 mM in a buffer containing 50 mM sodium phosphate and 50 mM NaCl (pH 7.0). CD spectra were collected at room temperature using a 0.1-mm path-length quartz cuvette over a wavelength range of 190–250 nm on a BioLogic MOS-500 spectrometer. All spectra were baseline-corrected against their respective buffer blanks. The secondary structure content of Ler^1–42^ was estimated using the BeStSel web server,^26^ with the deconvolution analysis restricted to the 200–250 nm range to avoid high-absorbance artifacts at elevated protein concentrations.

### F. SEC-MALS

Size-exclusion chromatography coupled with multi-angle light scattering (SEC-MALS) was performed to determine the absolute molecular weights of Ler constructs. Samples of Ler^1–36^, Ler^1–39^, Ler^1–42^, and Ler^18–74^ (100 *μ*L at 4 mg/mL) were prepared in a buffer containing 50 mM sodium phosphate, 50 mM NaCl, and 0.05% NaN3 (pH 7.0). Samples were individually loaded onto a Superdex 200 Increase 10/300 GL column connected to an AKTA purification system (GE Healthcare) that was equilibrated with the same buffer. The system was coupled to a DAWN HELEOS static light scattering detector (Wyatt Technology) operating at a laser wavelength of 658 nm with detection angles of 45°, 90°, and 135°, as well as a refractive index (RI) detector for concentration determination. Data acquisition and analysis were performed using ASTRA software (Wyatt Technology).

### G. Chemical cross-linking

Chemical cross-linking experiments were performed using ethylene glycol bis (succinimidyl succinate) (EGS) (Pierce). Protein samples (Ler^18–74^, Ler^1–36^, Ler^1–39^, and Ler^1–42^) were prepared at a concentration of 100 *μ*M in a buffer containing 50 mM sodium phosphate and 50 mM NaCl (pH 7.0). Cross-linking reactions were initiated by mixing the protein solutions with varying concentrations of EGS and incubating at room temperature (25 °C) for 15 min. The reactions were quenched by the addition of 1 M Tris-HCl to a final concentration of 100 mM. The reaction products were subsequently analyzed by SDS-PAGE and Coomassie brilliant blue staining.

### H. Partial proteolysis

Ler^1–74^ (2 mg/mL) was incubated with varying concentrations of Proteinase K at 4 °C for 1 h. The reaction products were separated and analyzed by SDS-PAGE. The prominent protease-resistant bands, migrating slightly below the intact Ler^1–74^ construct, were excised from the gel. To identify the sequence coverage of these resistant fragments, the excised bands were subjected to standard in-gel tryptic digestion, and the resulting peptides were subsequently analyzed using a high-field Orbitrap mass spectrometer (Orbitrap Elite, Thermo Fisher) at the Analytical Instrumentation Center of Peking University.

## III. RESULTS

### A. Ler has two distinct dimerization interfaces

**FIG. 1.**
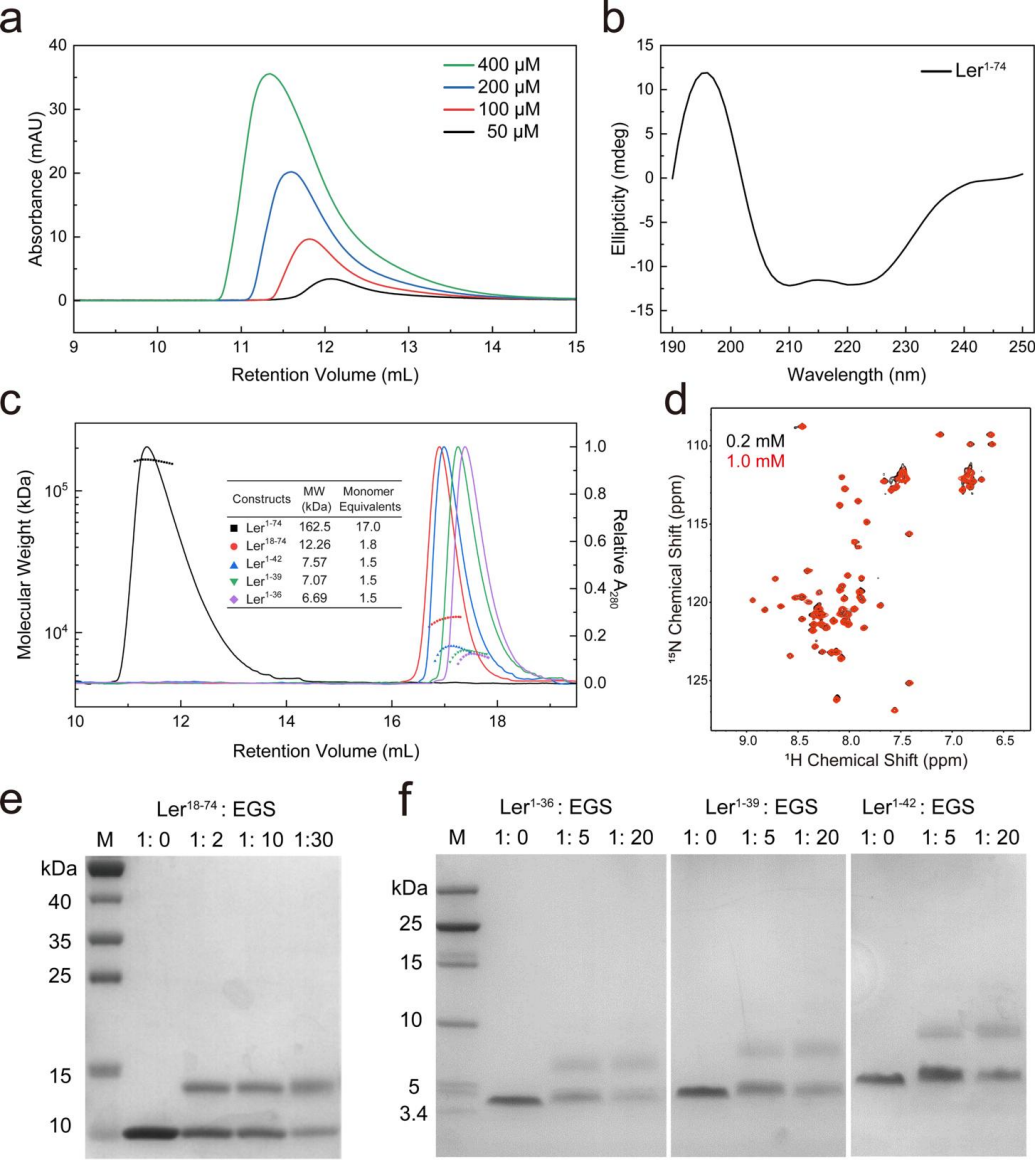
Characterization of the oligomerization domain of Ler. (a) Size exclusion chromatography (SEC) profiles of Ler^1–74^. Protein samples were loaded on a Superdex 200 Increase 10/300 GL column at concentrations of 50 *μ*M (black), 100 *μ*M (red), 200 *μ*M (blue), and 400 *μ*M (green), with an injection volume of 100 *μ*L. (b) Circular dichroism of Ler^1–74^ (1.0 mg/mL), exhibiting characteristic minima at 208 and 222 nm indicative of a predominantly α-helical structure. (c) SEC-MALS analysis of Ler constructs [Ler^1–74^ (black), Ler^18–74^ (red), Ler^1–42^ (blue), Ler^1–39^ (green), and Ler^1–36^ (purple)]. Solid lines represent the normalized UV absorbance at 280 nm (right axis), while the scattered points indicate the calculated molecular mass (left axis). The color of scattered points corresponds to the UV trace of the respective construct. The inset table summarizes the measured molecular weights (MW) and monomer equivalents. The monomer equivalents were derived using theoretical monomer molecular weights of 9.6 kDa for Ler^1–74^, 7.0 kDa for Ler^18–74^, 5.2 kDa for Ler^1–42^, 4.8 kDa for Ler^1–39^, and 4.5 kDa for Ler^1–36^. (d) Overlay of 2D ^1^H-^15^N HSQC spectra of Ler^18–74^ acquired at protein concentrations of 0.2 mM (black) and 1.0 mM (red). (e) Chemical cross-linking of Ler^18–74^ with EGS. The protein was incubated with increasing concentrations of EGS and analyzed by SDS-PAGE, showing a dominant dimer band. (f) Chemical cross-linking of N-terminal fragments Ler^1–36^, Ler^1–39^, and Ler^1–42^ with EGS. Dimer bands are observed for these constructs.

To characterize the oligomerization of Ler, we have expressed residues 1–74 of Ler (Ler^1–74^), including the N-terminal oligomerization domain and the interdomain linker region but excludes the C-terminal DNA-binding domain covers residues 75–111.^14^ Circular dichroism (CD) indicates that Ler^1–74^ is predominantly α-helical [Fig. 1(b)], which aligns with secondary structure predictions from both PSIPRED^27,28^ and previous work by Mellies *et al.*^8,15^ Size exclusion chromatography analysis indicates that Ler^1–74^ forms high order multimers, with the molecular size of the multimer slightly dependent on the protein concentration [Fig. 1(a)]. The estimated molecular weight is 164 kDa at 50 *μ*M from size exclusion chromatography analysis, while it is 212 kDa at 400 *μ*M (Fig. S2). SEC-MALS determined the apparent molecular mass of Ler^1–74^ at 5 mg/mL to be 162.5 kDa, which corresponds to an oligomeric state of about 17 monomers (monomer molecular mass = 9.6 kDa).

We have tested whether Ler forms oligomers through two relatively independent dimer interfaces, as does H-NS.^16^ Limited proteolysis of Ler^1–74^ with proteinase K was carried out and mass spectrometric analysis of three proteinase-resistant fragments revealed tryptic peptides covering residues 29–44 for band 1, 7–54 for band 2, and 18–74 for band 3 (Fig. S3). We thus tried to express residues 7–74 and 18–74, and residues 18–74 (Ler^18–74^) construct exhibited excellent soluble expression, while residues 7–74 expressed as insoluble inclusion bodies. SEC-MALS analysis yielded a molecular mass equivalent to 1.8 times that of Ler^18–74^ monomer [Fig. 1(c)], consistent with a dimer, which is further confirmed from EGS chemical cross-linking experimental results [Fig. 1(e)]. 2D ^1^H-^15^N HSQC spectra indicate that Ler^18–74^ is well folded and has no concentration-dependent signal change [Fig. 1(d)].

We next generated and expressed N-terminal fragments containing residues 1–27, 1–30, 1–33, 1–36, 1–39, and 1–42, and only the last three constructs could be expressed in soluble form in *E. coli*. EGS chemical cross-linking experiments showed dimer bands of Ler^1–36^, Ler^1–39^, and Ler^1–42^ [Fig. 1(e)], and the apparent molecular masses determined by SEC-MALS were 1.5 times the monomeric mass [Fig. 1(c)].

**FIG. 2.**
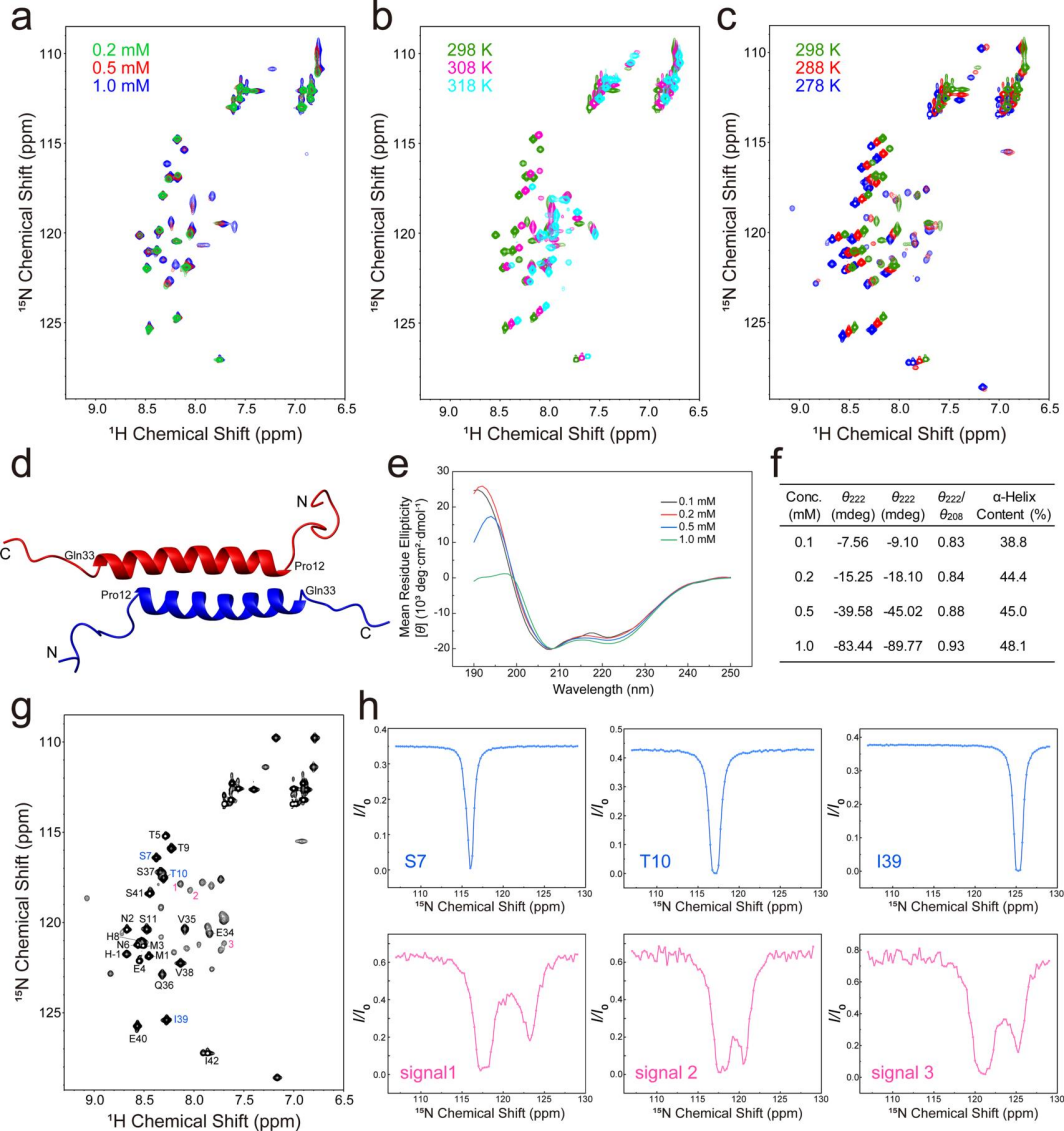
NMR and CD characterizations of Ler^1–42^. (a) Overlay of 2D ^1^H-^15^N HSQC spectra of Ler^1–42^ acquired at protein concentrations of 0.2 mM (green), 0.5 mM (red), and 1.0 mM (blue). New signals appear as the concentration increases, revealing a concentration-dependent dimerization. (b) Overlay of 2D ^1^H-^15^N HSQC spectra of Ler^1–42^ acquired at temperatures of 298 K (green), 308 K (magenta), and 318 K (cyan). Signals clearly disappear as the temperature increases. (c) Overlay of 2D ^1^H-^15^N HSQC spectra of Ler^1–42^ acquired at temperatures of 298 K (green), 288 K (red), and 278 K (blue). New signals appear as the temperature decreases. (d) Structural model of Ler^1–40^ predicted by AlphaFold3. (e) Overlay of CD spectra of Ler^1–42^ acquired at protein concentrations of 0.1 mM (blank), 0.2 mM (red), 0.5 mM (blue), and 1.0 mM (green). (f) Summary of the *θ*_222_/*θ*_208_ ratio and the corresponding α-helical content of Ler^1–42^ at different protein concentrations. (g) 2D ^1^H-^15^N HSQC spectrum of Ler^1–42^ with NMR-signal assignments indicated by single-letter codes and residue numbers. Residues highlighted in blue (S7, T10, I39) and unassigned signals labeled in pink (1, 2, 3) correspond to the respective CEST profiles presented in (e). (h) ^15^N-CEST profiles of the residues highlighted in blue and pink in (d). Top panels (blue): Residues flanking the dimerization interface exhibit single intensity dips, indicating the absence of slow chemical exchange. Bottom panels (pink): Unassigned residues located at the interface show distinct double intensity dips, revealing the presence of conformational exchange on the millisecond timescale.

2D ^1^H-^15^N HSQC spectra of Ler^1–36^, Ler^1–39^, and Ler^1–42^ display significantly fewer signals than the number of residues in each construct (Fig. S4), where most NH signals of Ler^1–36^ and Ler^1–39^ can overlap with those of Ler^1–42^. At room temperature, there are less than 20 NH signals in 2D ^1^H-^15^N HSQC spectrum of Ler^1–42^ at 0.1 mM concentration, while more NH signals can be observed at higher concentrations [Fig. 2(a)], suggesting that Ler^1–42^ exhibits concentration-dependent dimerization. The number of NH signals of Ler^1–42^ is also temperature-dependent, with signals disappeared at higher temperature [Fig. 2(b)] and more signals appeared at lower temperature [Fig. 2(c)]. The NMR signal missing at lower concentration or higher temperature should be resulted from the monomer/dimer exchange rates at intermediate NMR timescale. At 278 K, there are about 40 NH signals observed, consistent with number of NH groups of Ler^1–42^, and all newly appeared signals are relatively weak in intensity [Fig. 2(c)]. We tried to get backbone NMR resonance assignments of Ler^1–42^ with a 1 mM sample at 278 K, based on 3D triple resonance experimental data. However, we were only able to unambiguously assign residues 1–11 and 34–42 [Fig. 2(d)], corresponding to those signals appeared in the spectrum of a 0.1 mM sample at 298 K. These should suggest that residues 12–33 should form a dynamic dimer interface. Consistently, structure models generated by AlphaFold3 for residues 1–40 of Ler reveal that residues 12–33 can form an anti-parallel coiled coil dimer [Fig. 2(e)]. Indeed, circular dichroism (CD) analysis revealed a typical α-helical spectrum for Ler^1–42^, characterized by two minima at 208 and 222 nm [Fig. 2(f)]. Deconvolution of the spectra using the BeStSel server^26^ estimated the α-helix content to range from ∼39% to 48%, progressively increasing with protein concentration [Fig. 2(g)]. Furthermore, the ellipticity ratio between 222 and 208 nm (*θ*_222_/*θ*_208_)increased from 0.83 to 0.93 as the protein concentration increased from 0.1 to 1 mM [Fig. 2(g)], which is an indication of the formation of coiled coil dimer from isolated α-helices at higher concentrations.^29–31^ We thus designated this dimeric interface as dimer Site-1, demonstrating that its dimerization is dependent on both protein concentration and temperature.

To directly probe the stability and dynamics of this interface, we performed chemical exchange saturation transfer (CEST) experiments at 278 K. The CEST profiles for residues flanking the predicted interface (e.g., S7, T10, I39) showed a single dip, characteristic of nuclei in a single major conformational state [Fig. 2(e), top]. In contrast, the profiles for unassigned signals display clearly at least two major dips, providing direct evidence for conformational exchange on the millisecond timescale between two distinct states [Fig. 2(e), bottom]. Thus, the low-temperature condition does not abolish the underlying dynamics of the Site-1 dimer interface.

### B. Solution structure of Ler^18–74^ dimer

**FIG. 3.**
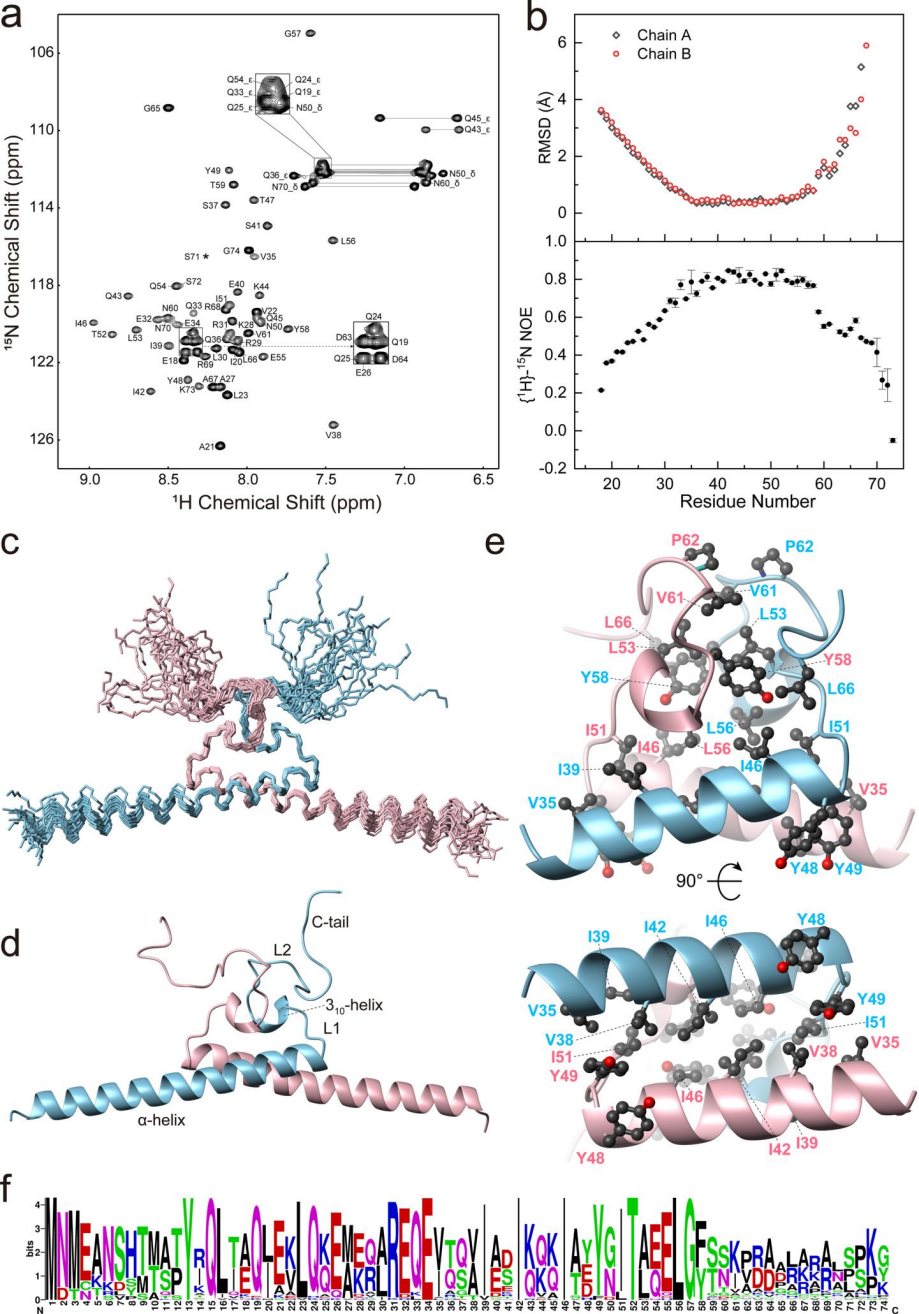
Solution structure of Ler^18–74^ dimer. (a) 2D ^1^H-^15^N HSQC spectrum of Ler^18–74^ with NMR signal assignments indicated by single-letter codes and residue numbers. Side-chain NH_2_ peaks of Q and N are labeled with *ε* and *δ*, respectively. Asterisks (*) denote residues with signals below the plotting threshold. (b) Top panel: Per-residue backbone heavy atom RMSD values for the two chains of the Ler^18–74^ dimer in the 20-structure ensemble. Bottom panel: Heteronuclear {^1^H}-^15^N NOE values of Ler^18–74^. (c) Superposition of backbone trace of Ler^18–74^ dimer in the ensemble of the 20 lowest-energy structures. The two chains are colored in sky blue and pink, respectively. (d) Ribbon representation of the representative lowest-energy structure of the Ler^18–74^ dimer. (e) Close-up view of the hydrophobic core of Site-2 dimer interface. Side chains of residues involved in the dimer interface are shown as ball-and-stick. (f) Sequence logo of the Ler family proteins (residues 1–74). Homologous sequences were retrieved from the NCBI RefSeq database using BLAST with Ler^1–74^ as the query. Only sequences sharing 40%–95% identity were selected to generate the logo using the WebLogo program.^32^

We proceeded to determine the solution structure of Ler^18–74^. Nearly complete backbone chemical shift assignments were achieved for the Ler^18–74^ dimer [Fig. 3(a)]. Overall, we obtained assignments for 97% of the backbone and over 90% of side chain resonances. The solution structure of the Ler^18–74^ dimer was determined based on a total of 5898 NOE-derived distance restraints, with 684 intermolecular NOE restraints (Fig. S5) from a hybrid-labeled Ler^18–74^ sample generated by refolding of urea denatured ^15^N-^13^C-labeled and unlabeled Ler^18–74^ (1:1) protein mixture (Fig. S4). The ensemble of the 20 lowest-energy AMBER structures [Fig. 3(c)] converged with a root mean square deviation (RMSD) of 0.35 Å for backbone heavy atoms (residues 33–58) relative to the mean structure. Table I summarizes the structural statistics and refinement restraints.

**TABLE I.** Restraints and structural statistics for Ler^18–74^ dimer.

Restraints	
Total NOE	5898
Intra-residue	1354
Inter-residue	2260
Sequential (|*i* – *j*| = 1)	890
Nonsequential (|*i* – *j*| > 1)	1370
Ambiguous	2284
Hydrogen bonds	82
Dihedral angle restraints	204
φ angle	116
ψ angle	88
Structure statistics	
Violations	
Distance restraints (> 0.2 Å)	0
Dihedral angle restraints (> 5°)	0
RMSD to mean structure (Å) (residues 33–58)	
All heavy atoms	0.83
Backbone heavy atoms	0.35
PROCHECK	
Most favored regions (%)	95.4
Additionally allowed regions (%)	4.6
Generously allowed regions (%)	0
Disallowed regions (%)	0

The Ler^18–74^ dimer is assembled in an anti-parallel tail-to-tail manner, with each protomer consisting of a long α-helix (residues 18–48) and a 3_10_-helix (residues 53–56) [Fig. 3(d)]. The dimer interface is mainly composed of residues 35–66 (designated as dimer Site-2), consisting the second half of the α-helix and the 3_10_-helix, along with two ordered loops (L1: residues 49–52; L2: residues 57–66) [Fig. 3(d)]. Residues 35–48 of the two α-helices form antiparallel coiled coil with the sidechains of residue I42 contacting each other [Fig. 3(e)]. The side chain of Y48, the last residue of the α-helix, is stacking to that of the adjacent Y49 on L1 loop, which make hydrophobic contact with side chains of residues V35 and V38 on the other protomer [Fig. 3(e)]. Residue I51 on L1 loop also participates hydrophobic interactions with residue V35, V38, and I42 of the other protomers [Fig. 3(e)]. The two 3_10_-helices are positioned on top of the coiled coil, with the side chains of residue L56 contacting preceding residue I51 and residue I42 of the other protomer. The following L2 loops cover the two 3_10-_helices, with inter-chain contacts between side chains of the two V61 and between residues Y58 and L66. The side chain of L53 on the 3_10_-helix also contacts side chains of Y58 and L61 on the L2 loop of the other molecule [Fig. 3(e)]. As a result, a large hydrophobic core is formed, which confers the stability of Site-2 dimer. The dimer interface is further stabilized by four intermolecular hydrogen bonds, formed between the H^ε2^ of Q43 and carbonyl oxygen of E55, and between the hydroxyl of Y58 and the main-chain carbonyl oxygen of I51. Additionally, four intramolecular hydrogen-bonds formed between backbone amide and carbonyl groups (Asn50-H^N^…Thr47-O, Ile51-H^N^…Ile46-O, Val61-H^N^…Tyr58-O', Leu66-H^N^…Asp64-O) also contribute to the folding of the dimer interface.

In the ensemble of 20 structures, per-residue backbone heavy atoms RMSD values of residues 31–58 are all below 1 Å, while the other residues are above 1 Å [Fig. 3(b), top]. Although residues 59–66 are relatively ordered and the hydrophobic side chains of residues V61 and L66 are involved in intermolecular interactions at the edges of the hydrophobic core, the corresponding RMSD values become larger, 1.3–1.8 Å for residues 59–62 and 2.1–3.7 Å for residues 63–66 [Fig. 3(b), top]. Consistently, the heteronuclear {^1^H}-^15^N NOE values for residues 31–58 are all above 0.65, while those of residues 59–66 are between 0.50 and 0.63 [Fig. 3(b), bottom]. The structural flexibility starts to increase significantly at residue T59, but decreased slightly at L66. The N-terminal residues 18–30 show heteronuclear {^1^H}-^15^N NOE values gradually increased from 0.21 to 0.64, indicating flexibility for the first half of the two α-helices [Fig. 3(b), bottom]. Sequence alignment logo of the primary sequences of Ler family proteins also reveals that residues 59–74 are much less conserved, while the preceding residues L56, G57, and Y(F)58 are highly conserved [Fig. 3(f)].^32^ These suggest that residues 59–66 should play a lesser role in the dimerization of Site-2. Searches of the Protein Data Bank using the Dali^33^ or PDBePISA^34^ servers did not identify any structures similar to the dimer interface of Ler^18–74^, and the top output structures from both servers are mainly coiled coil dimer structures.

To validate the functional importance of Site-2, mutations Y49A, L56A, and Y58A within the hydrophobic core of Site-2 were individually introduced into Ler^1–74^. These mutants were either insolubly expressed or precipitated during purification, demonstrating that disrupting the Site-2 hydrophobic core is catastrophic for the correct folding and stability of the entire N-terminal oligomerization domain.

## IV. DISCUSSION

More than two decades after initial discovery of Ler protein, and despite extensive research into its functional properties, the structure–function relationship underlying Ler's mechanism of action remains poorly understood. In particular, the detailed mechanism of Ler oligomerization has not been elucidated, and the critical differences between its assembly mechanism and that of H-NS remain a key unresolved question. Our study reveals that the oligomerization domain (residues 1–66) of Ler is also organized with two distinct dimerization interfaces, with Site-1 interface consisting of residues 12–33 and Site-2 interface covering residues 35–66. There is a short linker (residues 67–74) between dimer Site-2 and the DNA binding domain, and the N-terminal 11 residues should be flexible in Ler.

**FIG. 4.**
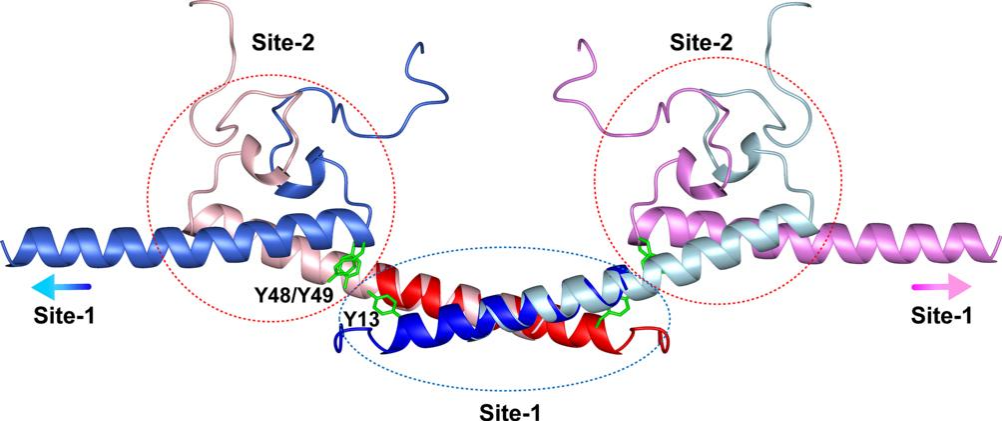
Proposed structural model for the continuous oligomerization of Ler. The model was generated by superimposing the overlapping residues (20–33) of two dimer structures (pink/royal blue and light blue/violet) of Ler^18–74^ onto the AlphaFold3 predicted dimer structure (red and blue) of Ler^1–34^. Site-1 and Site-2 dimer interfaces are indicated by blue and red dash lines. Side chains of the conserved residues Y13, Y48, and Y49 are shown in green sticks. The blue and purple gradient arrows indicate the positions of subsequent Site-1 dimer interfaces extending outward, illustrating the mechanism of continuous oligomerization.

We have determined the solution structure of the Site-2 dimer, which is a stable anti-parallel dimer. However, the dimerization of Site-1 interface alone is very dynamic, highly dependent on the protein concentration and temperature, which is consistent with the concentration-dependent multimerization behavior of the oligomerization domain of Ler. As mentioned above, AlphaFold3 predicted that residues 12–33 adopt an anti-parallel coiled coil dimer, with side chains of residues Y13, L16, I20, L23, A27, L30, and R31 participating intermolecular hydrophobic interactions [Fig. 2(d)]. We thus generated a structure model to illustrate the N-terminal oligomerization domain by fitting residues 20–33 of one protomer of the Ler^18–74^ dimer structure (Site-2) onto the corresponding residues of each molecule of the AlphaFold3-predicted structure of Ler^1–34^ (Fig. 4). The resulting model indicates the two dimer interfaces can enable Ler to multimerize through the combination of Site-1 head-to-head and Site-2 tail-to-tail dimers, similar to that H-NS oligomerizes through two independent dimer interfaces.^35^

In the structure model, the conserved residue Y13 in Site-1 dimer is positioned close to Y49 and Y50 of the Site-2 dimer, and it is possible that Site-1 dimer is stabilized in the intact oligomerization domain of Ler due to interaction with Site-2 (Fig. 4). This can explain that Ler^1–74^ only show weak concentration-dependent oligomerization behavior, quite different from the multimerization of H-NS, which is highly sensitive to temperature and concentration.^36^ The two dimer sites of the N-terminal oligomerization domain of H-NS are distant and relatively independent, and it has been shown that the N-terminal first dimer interface is stable, while the second dimer interface tends to unfold at human body temperature.^10^

It is well established for H-NS that dimers predominate at low concentration, while oligomer formation becomes favored in the micromolar range.^11,37^ Physiological intracellular concentrations (∼10–20 *μ*M) strongly favor multimerization.^11,38^ Single-molecule studies show that H-NS polymerizes along DNA in a concentration-dependent manner, forming rigid nucleoprotein filaments.^39^ It has been shown that environmental factors (temperature shifts, osmotic stress, pH) can alter oligomerization and DNA association of H-NS, and thus regulate the function of H-NS.^10,11,36,40,41^ In addition, it was also found that Lon protease can regulate the function of H-NS through proteolytic degradation and thus reducing its intracellular abundance to derepress foreign genes during infection.^42^ As an anti-silencing protein to counteract xenogeneic silencing of H-NS, it is still not clear whether the concentration or temperature-dependent oligomerization behaviors play a role in Ler's biological function. However, it is worth mentioning that Ler itself is subject to transcriptional autoinhibition, binding directly to its own promoter region to attenuate its transcription.^7^ This autorepression was observed even in the absence of H-NS and thus is intrinsic to Ler, indicating that the expression level of Ler needs to be controlled and fine-tuned for Ler to properly function. Additionally, it was found that Ler binding can result in DNA folding at lower concentrations, but not at higher concentrations; instead, it increases the DNA bending rigidity.^43^

The predicted Site-1 dimer interface of and the Site-2 dimer interface revealed by the solution structure of Ler^18–74^ are consistent with previous reports that mutants I20T, L23R, I42T, and I46T result are completely or partially defective in derepression of LEE5p.^44^ Also, it has been showed that only L56P mutant of Ler cannot coprecipitate with His-tagged WT Ler in a previous pull-down-based experiment, but not L16P and L23R mutants, even though all three mutants are defect in autorepression of LEE1 promoter and showing low binding affinity to the LEE1 and LEE2 regulatory regions.^13^ These results agree with that only L56P can affect the stable Site-2 dimer interface of Ler, while L16P and L23R mutations only impact the dynamic Site-1 dimer interface.^13^ A truncation mutant containing N-terminal 64 residues of Ler was reported to mediate a dominant-negative effect on the wild-type Ler, but not that with only N-terminal 49 residues, also consistent with the Site-2 dimer structure.^15^ As L66 is only loosely packed again Y58 in the solution structure of Ler^18–74^, it is likely not very critical for the dimerization of Site-2. Insertions of 11 or 6 alanine residues between residues N60 and V61 were found to impair the ability of Ler to increase LEE5 transcription,^15^ indicating the importance of V61 in the oligomerization of Ler.

We also tried to model the multimer structures of the Ler oligomerization domain on AlphaFold3 server (https://alphafoldserver.com/),^45^ and the resulting structure models are dependent on number of molecules (Fig. S7). If the number is less than 12, AlphaFold3 generally produce extended structures that with the two dimer interfaces propagating linearly. For 12 or more molecules, AlphaFold3 tends to produce closed ring structures, with the linker region positioned outside the ring for 12-mer, but inside the ring for 13-mer and above. It is the same case for full-length Ler, and AlphaFold3 predicts closed ring structures for 10-mer and above, all with the linker positioned inside the ring, while extended structures are generated for those with less than 10 molecules. These AlphaFold3 predicted structure models are not compatible with previous reports that free Ler protein should adopt compact globular shapes from atomic force microscopy and transmission electron microscopy analyses.^8,9^ More future studies are needed to reveal the oligomerization structure of Ler.

## SUPPLEMENTARY MATERIAL

See the supplementary material for additional details on the biochemical and structural analyses of Ler (Figure S1–S7).

## Data Availability

The atomic coordinates and structural restraints for the Ler^18-74^ dimer have been deposited in the Protein Data Bank (PDB) under the accession code 9XUZ. The NMR chemical shift assignments have been deposited in the Biological Magnetic Resonance Data Bank (BMRB) under the accession number 36813. The data that support the findings of this study are available within the article and its supplementary material.

## References

[1] J. B. Kaper, J. P. Nataro, and H. L. Mobley, “Pathogenic *Escherichia coli*,” Nat. Rev. Microbiol. 2(2), 123–140 (2004). 10.1038/nrmicro818 15040260

[2] J. L. Mellies, A. M. Barron, and A. M. Carmona, “Enteropathogenic and enterohemorrhagic *Escherichia coli* virulence gene regulation,” Infect. Immun. 75(9), 4199–4210 (2007). 10.1128/IAI.01927-06 17576759 PMC1951183

[3] J. L. Mellies, S. J. Elliott, V. Sperandio, M. S. Donnenberg, and J. B. Kaper, “The Per regulon of enteropathogenic *Escherichia coli*: Identification of a regulatory cascade and a novel transcriptional activator, the locus of enterocyte effacement (LEE)-encoded regulator (Ler),” Mol. Microbiol. 33(2), 296–306 (1999). 10.1046/j.1365-2958.1999.01473.x 10411746

[4] V. Sperandio, J. L. Mellies, R. M. Delahay, G. Frankel, J. A. Crawford, W. Nguyen, and J. B. Kaper, “Activation of enteropathogenic *Escherichia coli* (EPEC) LEE2 and LEE3 operons by Ler,” Mol. Microbiol. 38(4), 781–793 (2000). 10.1046/j.1365-2958.2000.02168.x 11115113

[5] S. J. Elliott, V. Sperandio, J. A. Girón, S. Shin, J. L. Mellies, L. Wainwright, S. W. Hutcheson, T. K. McDaniel, and J. B. Kaper, “The locus of enterocyte effacement (LEE)-encoded regulator controls expression of both LEE- and non-LEE-encoded virulence factors in enteropathogenic and enterohemorrhagic *Escherichia coli*,” Infect. Immun. 68(11), 6115–6126 (2000). 10.1128/IAI.68.11.6115-6126.2000 11035714 PMC97688

[6] A. Bhat, M. Shin, J. H. Jeong, H. J. Kim, H. J. Lim, J. H. Rhee, S. Y. Paik, K. Takeyasu, T. Tobe, H. Yen, G. Lee, and H. E. Choy, “DNA looping-dependent autorepression of LEE1 P1 promoters by Ler in enteropathogenic *Escherichia coli* (EPEC),” Proc. Natl. Acad. Sci. U. S. A. 111(25), E2586–2595 (2014). 10.1073/pnas.1402632111 24920590 PMC4078829

[7] T. Berdichevsky, D. Friedberg, C. Nadler, A. Rokney, A. Oppenheim, and I. Rosenshine, “Ler is a negative autoregulator of the LEE1 operon in enteropathogenic *Escherichia coli*,” J. Bacteriol. 187(1), 349–357 (2005). 10.1128/JB.187.1.349-357.2005 15601719 PMC538822

[8] J. L. Mellies, G. Benison, W. McNitt, D. Mavor, C. Boniface, and F. J. Larabee, “Ler of pathogenic *Escherichia coli* forms toroidal protein-DNA complexes,” Microbiology 157(Pt 4), 1123–1133 (2011). 10.1099/mic.0.046094-0 21212119 PMC3139439

[9] J. García, T. N. Cordeiro, M. J. Prieto, and M. Pons, “Oligomerization and DNA binding of Ler, a master regulator of pathogenicity of enterohemorrhagic and enteropathogenic *Escherichia coli*,” Nucl. Acids Res. 40(20), 10254–10262 (2012). 10.1093/nar/gks846 22965122 PMC3488262

[10] X. Zhao, U. F. Shahul Hameed, V. Kharchenko, C. Liao, F. Huser, J. M. Remington, A. K. Radhakrishnan, M. Jaremko, Ł. Jaremko, S. T. Arold, and J. Li, “Molecular basis for the adaptive evolution of environment-sensing by H-NS proteins,” eLife 10, e57467 (2021). 10.7554/eLife.57467 33410747 PMC7817174

[11] B. Lukose, T. Maruno, M. A. Faidh, S. Uchiyama, and A. N. Naganathan, “Molecular and thermodynamic determinants of self-assembly and hetero-oligomerization in the enterobacterial thermo-osmo-regulatory protein H-NS,” Nucl. Acids Res. 52(5), 2157–2173 (2024). 10.1093/nar/gkae090 38340344 PMC10954469

[12] R. S. Winardhi, J. Yan, and L. J. Kenney, “H-NS regulates gene expression and compacts the nucleoid: Insights from single-molecule experiments,” Biophys. J. 109(7), 1321–1329 (2015). 10.1016/j.bpj.2015.08.016 26445432 PMC4601063

[13] G. Yerushalmi, C. Nadler, T. Berdichevski, and I. Rosenshine, “Mutational analysis of the locus of enterocyte effacement-encoded regulator (Ler) of enteropathogenic *Escherichia coli*,” J. Bacteriol. 190(23), 7808–7818 (2008). 10.1128/JB.00663-08 18835988 PMC2583616

[14] T. N. Cordeiro, H. Schmidt, C. Madrid, A. Juárez, P. Bernadó, C. Griesinger, J. García, and M. Pons, “Indirect DNA readout by an H-NS related protein: Structure of the DNA complex of the C-terminal domain of Ler,” PLoS Pathog. 7(11), e1002380 (2011). 10.1371/journal.ppat.1002380 22114557 PMC3219716

[15] J. L. Mellies, F. J. Larabee, M. A. Zarr, K. L. Horback, E. Lorenzen, and D. Mavor, “Ler interdomain linker is essential for anti-silencing activity in enteropathogenic *Escherichia coli*,” Microbiology 154(Pt 12), 3624–3638 (2008). 10.1099/mic.0.2008/023382-0 19047730 PMC2659682

[16] S. T. Arold, P. G. Leonard, G. N. Parkinson, and J. E. Ladbury, “H-NS forms a superhelical protein scaffold for DNA condensation,” Proc. Natl. Acad. Sci. U. S. A. 107(36), 15728–15732 (2010). 10.1073/pnas.1006966107 20798056 PMC2936596

[17] F. Delaglio, S. Grzesiek, G. W. Vuister, G. Zhu, J. Pfeifer, and A. Bax, “NMRPipe: A multidimensional spectral processing system based on UNIX pipes,” J. Biomol. NMR 6(3), 277–293 (1995). 10.1007/BF00197809 8520220

[18] B. A. Johnson and R. A. Blevins, “NMR View: A computer program for the visualization and analysis of NMR data,” J. Biomol. NMR 4(5), 603–614 (1994). 10.1007/BF00404272 22911360

[19] Y. Shen and A. Bax, “Protein backbone and sidechain torsion angles predicted from NMR chemical shifts using artificial neural networks,” J. Biomol. NMR 56(3), 227–241 (2013). 10.1007/s10858-013-9741-y 23728592 PMC3701756

[20] B. M. Duggan, G. B. Legge, H. J. Dyson, and P. E. Wright, “SANE (structure assisted NOE evaluation): An automated model-based approach for NOE assignment,” J. Biomol. NMR 19(4), 321–329 (2001). 10.1023/A:1011227824104 11370778

[21] P. Güntert, “Automated NMR structure calculation with CYANA,” Methods Mol. Biol. 278, 353–378 (2004). 10.1385/1-59259-809-9:353 15318003

[22] P. Güntert, C. Mumenthaler, and K. Wüthrich, “Torsion angle dynamics for NMR structure calculation with the new program DYANA,” J. Mol. Biol. 273(1), 283–298 (1997). 10.1006/jmbi.1997.1284 9367762

[23] D. A. Case, I. Y. Ben-Shalom, S. R. Brozell, D. S. Cerutti, T. E. Cheatham III, V. W. D. Cruzeiro, T. A. Darden, R. E. Duke, D. Ghoreishi, M. K. Gilson, H. Gohlke, A. W. Goetz, D. Greene, R. Harris, N. Homeyer, Y. Huang, S. Izadi, A. Kovalenko, T. Kurtzman, T. S. Lee, S. LeGrand, P. Li, C. Lin, J. Liu, T. Luchko, R. Luo, D. J. Mermelstein, K. M. Merz, Y. Miao, G. Monard, C. Nguyen, H. Nguyen, I. Omelyan, A. Onufriev, F. Pan, R. Qi, D. R. Roe, A. Roitberg, C. Sagui, S. Schott-Verdugo, J. Shen, C. L. Simmerling, J. Smith, R. Salomon-Ferrer, J. Swails, R. C. Walker, J. Wang, H. Wei, R. M. Wolf, X. Wu, L. Xiao, D. M. York, and P. A. Kollman, *Amber 2018* (University of California, San Francisco, CA, 2018).

[24] R. A. Laskowski, J. A. Rullmannn, M. W. MacArthur, R. Kaptein, and J. M. Thornton, “AQUA and PROCHECK-NMR: Programs for checking the quality of protein structures solved by NMR,” J. Biomol. NMR 8(4), 477–486 (1996). 10.1007/BF00228148 9008363

[25] A. Shapiro, J. D. Botha, A. Pastore, and A. M. Lesk, “A method for multiple superposition of structures,” Acta Crystallogr. A 48(Pt 1), 11–14 (1992). 10.1107/S010876739100867X 1550663

[26] A. Micsonai, F. Wien, É. Bulyáki, J. Kun, É. Moussong, Y. H. Lee, Y. Goto, M. Réfrégiers, and J. Kardos, “BeStSel: A web server for accurate protein secondary structure prediction and fold recognition from the circular dichroism spectra,” Nucl. Acids Res. 46(W1), W315–w322 (2018). 10.1093/nar/gky497 29893907 PMC6031044

[27] D. T. Jones, “Protein secondary structure prediction based on position-specific scoring matrices,” J. Mol. Biol. 292(2), 195–202 (1999). 10.1006/jmbi.1999.3091 10493868

[28] D. W. A. Buchan and D. T. Jones, “The PSIPRED protein analysis workbench: 20 years on,” Nucl. Acids Res. 47(W1), W402–w407 (2019). 10.1093/nar/gkz297 31251384 PMC6602445

[29] T. M. Cooper and R. W. Woody, “The effect of conformation on the CD of interacting helices: A theoretical study of tropomyosin,” Biopolymers 30(7–8), 657–676 (1990). 10.1002/bip.360300703 2275971

[30] S. Y. Lau, A. K. Taneja, and R. S. Hodges, “Synthesis of a model protein of defined secondary and quaternary structure. Effect of chain length on the stabilization and formation of two-stranded alpha-helical coiled-coils,” J. Biol. Chem. 259(21), 13253–13261 (1984). 10.1016/S0021-9258(18)90686-1 6490655

[31] N. E. Zhou, C. M. Kay, and R. S. Hodges, “Synthetic model proteins. Positional effects of interchain hydrophobic interactions on stability of two-stranded alpha-helical coiled-coils,” J. Biol. Chem. 267(4), 2664–2670 (1992). 10.1016/S0021-9258(18)45932-7 1733963

[32] G. E. Crooks, G. Hon, J. M. Chandonia, and S. E. Brenner, “WebLogo: a sequence logo generator,” Genome Res. 14(6), 1188–1190 (2004). 10.1101/gr.849004 15173120 PMC419797

[33] L. Holm, “Dali server: Structural unification of protein families,” Nucl. Acids Res. 50(W1), W210–w215 (2022). 10.1093/nar/gkac387 35610055 PMC9252788

[34] E. Krissinel and K. Henrick, “Inference of macromolecular assemblies from crystalline state,” J. Mol. Biol. 372(3), 774–797 (2007). 10.1016/j.jmb.2007.05.022 17681537

[35] D. C. Grainger, “Structure and function of bacterial H-NS protein,” Biochem. Soc. Trans. 44(6), 1561–1569 (2016). 10.1042/BST20160190 27913665

[36] U. F. Shahul Hameed, C. Liao, A. K. Radhakrishnan, F. Huser, S. S. Aljedani, X. Zhao, A. A. Momin, F. A. Melo, X. Guo, C. Brooks, Y. Li, X. Cui, X. Gao, J. E. Ladbury, Ł. Jaremko, M. Jaremko, J. Li, and S. T. Arold, “H-NS uses an autoinhibitory conformational switch for environment-controlled gene silencing,” Nucl. Acids Res. 47(5), 2666–2680 (2019).Ł 10.1093/nar/gky1299 30597093 PMC6411929

[37] M. Falconi, M. T. Gualtieri, A. La Teana, M. A. Losso, and C. L. Pon, “Proteins from the prokaryotic nucleoid: Primary and quaternary structure of the 15-kD *Escherichia coli* DNA binding protein H-NS,” Mol. Microbiol. 2(3), 323–329 (1988). 10.1111/j.1365-2958.1988.tb00035.x 3135462

[38] T. Ali Azam, A. Iwata, A. Nishimura, S. Ueda, and A. Ishihama, “Growth phase-dependent variation in protein composition of the *Escherichia coli* nucleoid,” J. Bacteriol. 181(20), 6361–6370 (1999). 10.1128/JB.181.20.6361-6370.1999 10515926 PMC103771

[39] R. T. Dame, M. C. Noom, and G. J. Wuite, “Bacterial chromatin organization by H-NS protein unravelled using dual DNA manipulation,” Nature 444(7117), 387–390 (2006). 10.1038/nature05283 17108966

[40] R. A. van der Valk, J. Vreede, L. Qin, G. F. Moolenaar, A. Hofmann, N. Goosen, and R. T. Dame, “Mechanism of environmentally driven conformational changes that modulate H-NS DNA-bridging activity,” eLife 6, e27369 (2017). 10.7554/eLife.27369 28949292 PMC5647153

[41] S. Ono, M. D. Goldberg, T. Olsson, D. Esposito, J. C. Hinton, and J. E. Ladbury, “H-NS is a part of a thermally controlled mechanism for bacterial gene regulation,” Biochem. J. 391(Pt 2), 203–213 (2005). 10.1042/BJ20050453 15966862 PMC1276917

[42] J. Choi and E. A. Groisman, “Salmonella expresses foreign genes during infection by degrading their silencer,” Proc. Natl. Acad. Sci. U. S. A. 117(14), 8074–8082 (2020). 10.1073/pnas.1912808117 32209674 PMC7149492

[43] R. S. Winardhi, R. Gulvady, J. L. Mellies, and J. Yan, “Locus of enterocyte effacement-encoded regulator (Ler) of pathogenic *Escherichia coli* competes off histone-like nucleoid-structuring protein (H-NS) through noncooperative DNA binding,” J. Biol. Chem. 289(20), 13739–13750 (2014). 10.1074/jbc.M113.545954 24668810 PMC4022848

[44] S.-M. Choi, J.-H. Jeong, H. E. Choy, and M. Shin, “Amino acid residues in the Ler protein critical for derepression of the LEE5 promoter in enteropathogenic *E. coli*,” J. Microbiol. 54(8), 559–564 (2016). 10.1007/s12275-016-6027-6 27480636

[45] J. Abramson, J. Adler, J. Dunger, R. Evans, T. Green, A. Pritzel, O. Ronneberger, L. Willmore, A. J. Ballard, J. Bambrick, S. W. Bodenstein, D. A. Evans, C. C. Hung, M. O'Neill, D. Reiman, K. Tunyasuvunakool, Z. Wu, A. Žemgulytė, E. Arvaniti, C. Beattie, O. Bertolli, A. Bridgland, A. Cherepanov, M. Congreve, A. I. Cowen-Rivers, A. Cowie, M. Figurnov, F. B. Fuchs, H. Gladman, R. Jain, Y. A. Khan, C. M. R. Low, K. Perlin, A. Potapenko, P. Savy, S. Singh, A. Stecula, A. Thillaisundaram, C. Tong, S. Yakneen, E. D. Zhong, M. Zielinski, A. Žídek, V. Bapst, P. Kohli, M. Jaderberg, D. Hassabis, and J. M. Jumper, “Accurate structure prediction of biomolecular interactions with AlphaFold 3,” Nature 630(8016), 493–500 (2024). 10.1038/s41586-024-07487-w 38718835 PMC11168924

